# Metabolomics Applied to the Study of Extracellular Vesicles

**DOI:** 10.3390/metabo9110276

**Published:** 2019-11-12

**Authors:** Charles Williams, Mari Palviainen, Niels-Christian Reichardt, Pia R.-M. Siljander, Juan M. Falcón-Pérez

**Affiliations:** 1 Exosomes Laboratory, CIC bioGUNE, Bizkaia Technology Park, 48160 Derio, Spain; cwilliams@cicbiomagune.es; 2 Glycotechnology Laboratory, CIC biomaGUNE, Paseo Miramón 182, 20014 San Sebastián, Spain; nreichardt@cicbiomagune.es; 3 EV group, Molecular and Integrative Biosciences Research Programme, Faculty of Biological and Environmental Sciences, and CURED, Drug Research Program, Faculty of Pharmacy, Division of Pharmaceutical Biosciences, University of Helsinki, Viikinkaari 9, 00790 Helsinki, Finland; mari.palviainen@helsinki.fi (M.P.); pia.siljander@helsinki.fi (P.R.-M.S.); 4 EV-core, University of Helsinki, Viikinkaari 9, 00790 Helsinki, Finland; 5 CIBER-BBN, Paseo Miramón 182, 20014 San Sebastián, Spain; 6 Metabolomics Platform, CIC bioGUNE, Bizkaia Technology Park, 48160 Derio, Spain; 7 Centro de Investigación Biomédica en Red de Enfermedades Hepáticas y Digestivas (CIBERehd), 28029 Madrid, Spain; 8 IKERBASQUE Basque Foundation for Science, Bilbao, 48013 Bizkaia, Spain

**Keywords:** extracellular vesicles, exosomes, microvesicles, biomarkers, diagnostics, metabolic pathways

## Abstract

Cell-secreted extracellular vesicles (EVs) have rapidly gained prominence as sources of biomarkers for non-invasive biopsies, owing to their ubiquity across human biofluids and physiological stability. There are many characterisation studies directed towards their protein, nucleic acid, lipid and glycan content, but more recently the metabolomic analysis of EV content has also gained traction. Several EV metabolite biomarker candidates have been identified across a range of diseases, including liver disease and cancers of the prostate and pancreas. Beyond clinical applications, metabolomics has also elucidated possible mechanisms of action underlying EV function, such as the arginase-mediated relaxation of pulmonary arteries or the delivery of nutrients to tumours by vesicles. However, whilst the value of EV metabolomics is clear, there are challenges inherent to working with these entities—particularly in relation to sample production and preparation. The biomolecular composition of EVs is known to change drastically depending on the isolation method used, and recent evidence has demonstrated that changes in cell culture systems impact upon the metabolome of the resulting EVs. This review aims to collect recent advances in the EV metabolomics field whilst also introducing researchers interested in this area to practical pitfalls in applying metabolomics to EV studies.

## 1. Introduction

Extracellular vesicles (EVs) are bioactive nanosized vesicles that are secreted by cells that mediate intercellular communication through the transmission of functional biomolecules [1]. The term “EV” encompasses multiple designations as determined by the biogenetic pathway in question or biophysical characteristics of EVs [2]. Multivesicular-body-derived exosomes, for example, are created via the inward budding of endosomes, with the resulting intraluminal vesicles released through fusion of the endosome with the cell membrane. Elsewise, microvesicles directly bud off from the cell membrane and apoptotic bodies are the products of fragmented, dying cells. In addition to these three known classes of EV, uncharacterised subtypes are likely to exist differing in their composition, ultrastructure, or size [3,4]. Since the discovery in the mid-2000s that EVs can transfer functional mRNA [5,6], there have been increasing reports ascribing biological functions to EVs across an astounding range of contexts [1]. From developmental biology [7] and cardiovascular homeostasis [8] through to immunity [9] and angiogenesis [10], there appears to be a role for EVs in every instance where biological material is exchanged between cells. Correspondingly, the pathological dysregulation of these processes has also been reported in cancer [11], neurodegeneration [12], obesity [13] and inflammatory diseases [14], whilst the deployment of EVs by pathogens and microbiota is informing new paradigms on how we interact with microorganisms [15,16,17].

Consisting essentially of a lipid bilayer, EVs also comprise a repertoire of proteins, glycans and nucleic acids [1]. Some of these components are incorporated into vesicles during biogenesis and are thought to be characteristic markers of EVs. Notable examples include CD9, CD63 and CD81 of the tetraspanin protein family [18], high phosphatidylserine lipid content [19] and a conserved glycosylation signature that has been described for mammalian EVs [20]. Depending on the EV class in question, markers differ and guidelines from the Minimal information for studies of extracellular vesicles (MISEV) working group recommend that transmembrane/ glycosylphosphatidylinositol-anchored(GPI-anchored) proteins and cytosolic proteins be analysed in all bulk EV preparations in order to demonstrate the presence of EVs as well as non-EV structural proteins for purity control [21]. However, the comparatively limited repertoire of molecular markers cannot account for the diversity of functions ascribed to EVs, and online databases of EV omics studies show a vast assortment of EV-associated compounds beyond these [22,23], signifying other means of cargo packaging. Molecules may be trafficked directly to EVs through interactions with other biomolecules or indirectly packaged during vesicle formation processes that encapsulate pockets of cytosol [24]. Moreover, EV content is dynamic and shifts in response to perturbations in culture conditions [25]. As such, EVs present as reflections of their cellular source, and the analysis of vesicle content provides a nanoscopic window into the physiopathological state of said cells. There are various examples of this concept in action with the identification of EV protein, lipid and nucleic acid biomarkers for several diseases [26,27,28]. Additionally, EVs have been isolated from many human biofluids [1], engendering a huge interest in the use of EVs for non‑invasive liquid biopsies, and the EV diagnostics sector is projected to reach a value of $100 million by 2021 [29]. In recent years, several examples of metabolite biomarkers have been published [30,31] and EV metabolomics holds great promise against this backdrop.

Metabolites are described as any biologically relevant molecule <2 kDa in size. This comprises a large range of molecular species, from steroid hormones and lipids to the metabolic intermediates of nutrient anabolism and the monomers of the major biopolymer classes. Considering that all cellular processes involve metabolites in some form, assaying of metabolomes can provide information on any dysregulation to these in a manner analogous to using EVs to study cellular status. For example, UHPLC‑MS analysis of urinary EVs from prostate cancer patients identified abnormal levels of the steroid hormone dehydroepiandrosterone sulphate (DHEAS), suggesting this metabolite as a potential marker of prostate cancer and also as a means of monitoring disease progression [32]. Beyond providing disease biomarkers, metabolomics can also illuminate fundamental EV biology. It was shown that cancer-derived fibroblasts can supply nutrients to cancer cells through EVs, which further illuminates the mechanisms of the tumour niche [33]. Elsewise, EVs can also act as “metabolically active machines”, as proven by the metabolomic analysis of serum samples mixed with hepatocyte EVs. Therein, alterations in the levels of over 90 metabolites were detected, signifying the presence of functional enzymes in EVs [34].

**Figure 1:**
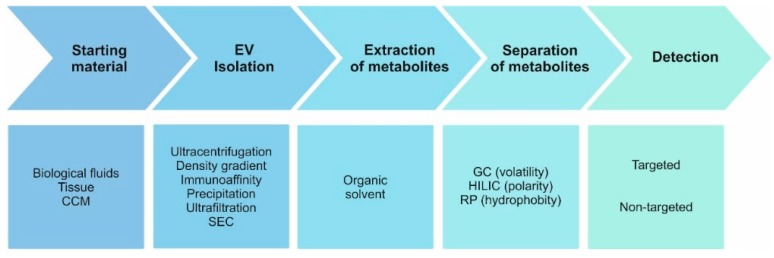
Key stages of the metabolomic analysis workflow for extracellular vesicle (EV) samples. Methodologies of EV isolation can impact the metabolome and should be carefully considered when comparing results from different studies. (CCM, cell conditioned medium; SEC, size exclusion chromatography; GC, gas chromatography; HILIC, hydrophilic interaction chromatography; RP, reversed-phase chromatography; Targeted analysis, detailed analysis of a predefined subset of the metabolome; Non-targeted analysis, maximum metabolite coverage).

Whilst EVs present exciting opportunities for metabolomics researchers, there are intractable practical difficulties inherent to working with EVs that must be considered from the outset of any potential projects—difficulties that mainly relate to the different classes of EVs and the heterogeneity within these. For example, whilst isolation protocols can be targeted towards exosomes, the physical and biomolecular properties of exosomes, microvesicles and apoptotic bodies overlap, meaning that any exosome-targeted preparation likely also contains other EVs as contaminants and it is difficult to assign vesicle designation with full certainty [21,35]. Even then, supposing totally pure exosome purification, the stochastic nature of vesicle biogenesis gives each exosome a potentially unique composition [2]. Any subsequent omics characterisation thus provides information for the bulk vesicle population and ignores the possible contribution of relevant subpopulations. EV subpopulations are also problematic for sample preparation, as multiple reports have shown how different isolation methods generate different results during downstream characterization [36,37,38,39]. The matrix from which EVs are obtained may partially dictate the isolation method, but the choice is further complicated by the increasing number of commercial and published EV isolation methods. Finally, production methods can also bias EV composition. This has been demonstrated for EVs from two prostate cancer cell lines produced from either standard culture flatware or from bioreactors, with the metabolic signature of these models shifting depending on the cell culture conditions [40]. Fortunately, despite these complexities, the actual metabolomic analysis of a purified EV sample is straightforward and does not present any sample-specific issues. In our hands, established techniques of metabolite extraction and analysis have proven sufficient in generating sufficient data. A workflow of possible methodologies for an EV metabolomics study is presented below (Figure 1).

Herein, we aim to cover the recent findings from EV metabolomics studies whilst placing an emphasis on the EV isolation and analysis methodologies used to highlight the best practices. The key studies so far and their salient information have been collected in Table 1 for easy reference. Consideration will also be given to the EV origins within studies, how the wider body of EV research can inform future EV metabolomics studies and whether a gold standard of EV sample preparation can ever be achieved. We will also cover how metabolomics of other sample types has been useful in understanding the role of EVs as metabolically active machines. It should be noted that there is crossover between EV metabolomics and lipidomics, as the size of biologically relevant lipids categorises them as metabolites. Since EV lipidomics is a more established field and has been excellently reviewed elsewhere [41,42], we have focused on metabolites as small polar molecules involved in cellular processes of metabolism.

## 2. Metabolomics of Patient-Derived EVs

Considering the potential of EVs for non-invasive liquid biopsies, the identification of robust metabolite biomarkers in EVs is a key goal. Such studies necessitate the use of EVs extracted from clinical samples for greater impact and translation. An instructive example comes from a metabolomics study of urinary EVs (uEVs) in the context of prostate cancer [32]. Therein, 50 mL of urine from patients with either prostate cancer (PCa) or benign prostatic hyperplasia (BPH) was sterile filtered and frozen at −80 °C immediately following collection. Subsequent EV isolation from the urine was performed by differential ultracentrifugation—first centrifuged for 30 min at 10,000 × *g* to remove the majority of larger vesicles before pelleting of small uEVs at 100,000 × *g* for 75 min. Processing of all uEV samples was performed by fractionation with methanol and chloroform into different phases of metabolites for UHPLC-MS analysis. Through this methodology, researchers detected differential metabolite content in the patient-derived uEVs and highlighted 76 of these as statistically significantly different between the PBH and PCa samples. Crucially, 3beta-hydroxyandors-5-en-17-one-3-sulphate (dehydroepiandrosterone sulphate, DHEAS) was raised in the uEVs from PCa patients compared to BHP patients, potentially enabling clinicians to distinguish between these often-confused diagnoses. The study continued with bioinformatics analysis of published gene expression data to identify a decrease in steroid sulphatase in the PCa samples, correlating with disease progression. DHEAS is a substrate for this enzyme, and its loss in advanced PCa suggests that DHEAS accumulation could also be used to stratify patients according to disease progression. The question remains as to whether a baseline DHEAS level could be established to show meaningful DHEAS deviations as a biomarker for initial PCa identification. Furthermore, the pathological relevance of EV-associated DHEAS is potentially interesting. No more than 10% of DHEAS is produced from the gonads of healthy males, instead primarily originating from the adrenal glands [51]. It is possible that the dysregulation of the steroid sulphatase gene in the cancer context could lead to more constitutive DHEAS signalling, exacerbating prostate tumour severity. In all, this study highlights how a combinatorial approach of metabolomics in combination with other methods can add value to diagnostics and provide new insights to disease.

Another proof-of-concept study for extracting EV metabolite biomarkers from biofluids was established with the plasma of endometrial adenocarcinoma (EAC) patients [43]. Plasma was obtained from 10 mL samples of blood from both EAC patients and control subjects before storage at −80 °C. EVs were again isolated through ultracentrifugation, although with a first centrifugation at 16,500× *g* and the second at 100,000× *g* for 120 min. Methanol–chloroform fractionation was employed to release metabolites and UPLC-ESI-MS was used for data acquisition. Due to inconsistencies between the data and metabolite databases, accurate mass-based identification of the detected metabolites could not be performed, beyond a few amino acids and substituted sugars. However, principal component analysis of metabolites revealed a clear separation between the plasma of healthy control subjects and those of the cancer patients, indicating the potential for even incomplete EV metabolomics in disease identification. Both these studies used ultracentrifugation for EV purification, albeit with some differences to their protocols. Previously described as the “gold standard” of EV isolation [52], this approach is straightforward and well suited to generating proof-of-concept data. However, ultracentrifugation is not appropriate for clinical diagnostics due to specific equipment requirements [53]. Other isolation methods are also available, such as size exclusion chromatography, immunoaffinity and precipitation-based methods. Each has their own advantages and drawbacks in terms of selectivity and yield, and these have been described with detail in a recent review [54]. In the case of urinary EVs, different clinically relevant EV purification methods were compared against ultracentrifugation, selected on the basis of minimal equipment requirement [37]. Said methodologies comprised several commercially available EV isolation kits working on precipitation principals and a method of lectin-based EV capture [55]. The protein and RNA composition of purified EVs was found to be heavily influenced by the isolation method, with EV markers CD9 and CD63 nearly absent in some purified samples. Inter-donor samples were also found to vary wildly in marker content when the same isolation method was used, highlighting the difficulty of establishing biomarker baselines. Considering this evidence, it is essential that this comparative approach is extended to EV metabolomics in order to ascertain whether there are similar method-dependent effects on EV metabolite content.

## 3. Metabolomics of EVs from Cell Culture

Whilst extracting EVs directly from biofluids has direct clinical prospects, there are advantages to culturing patient-derived cells in vitro for understanding disease. Specifically, the EVs of cancer-associated fibroblasts have been examined and a potential role found for their EVs in modulating the metabolism of cells from the cancerous PC3 line [33]. This study took fibroblasts originally isolated from cancer patient tumours and cultured these in either normal conditions or in media supplemented with various ^13^C-labeled molecules including glucose, pyruvate, and lysine. Therein, the commercial Total Exosome Isolation Reagent from Thermo Fisher was used to isolate EVs directly from the cell culture media and the study employed complementary metabolomics approaches to characterise the cargo of these EVs. GC-MS (gas chromatography) highlighted a high vesicular content of the nutrients pyruvate and citrate, whilst UHPLC identified a number of essential amino acids, with glutamine and arginine particularly enriched. Downstream functional studies proved that these metabolites, which were supplied by the EVs produced in the presence of ^13^C-labeled molecules, could be taken up and utilised by the receiving PC3 cells. The EVs were found to be within the size range for exosomes (30–100nm) according to nanoparticle tracking analysis, and were positive for CD63.

Beyond considerations in EV sample preparation, some forethought must also be given to the types of molecules one wishes to analyse. No single method is able to cover the entire spectrum of the metabolome. This is well exemplified with the EVs from red blood cells in relation to malaria and the transmission of the *Plasmodium falciparum* parasite by *Anopheles* spp. [44]. Understanding the processes of mosquito attraction to humans requires the study of volatile organic compounds as the transmitters of human scent over distances. Red blood cells from infected and non-infected volunteers were cultured ex vivo and the resulting conditioned media subjected to differential ultracentrifugation for 30 min at 15,000× *g* and 110,000× *g* for 70 min for EV purification. Metabolite extraction was performed concurrently with GC-MS using the headspace solid phase microextraction (HS-SPME) method. Briefly, EV samples were sealed in GC vials in the presence of a divinylbenzene/carboxen/polydimethylsiloxane fibre for 12 h at 37 °C before injection to the mass spectrometer. The HS-SPME GC-MS method was chosen to select for volatile organic compounds, and 18 of such were identified from EVs, of which diacetin was found to be increased in the EVs derived from infected red blood cells. The ultracentrifugation supernatants were also analysed, and in turn, hexanal was found solely in infected supernatants. Together, these are possible mosquito chemoattractants upregulated after infection with *P. falciparum*. which were discovered by metabolomics.

Importantly, the method of cell culture can also impact upon the metabolite composition of EVs, and may need to be considered when results of different studies are compared. This issue was highlighted by a recent article comparing the EVs of two prostate cancer cell lines produced either in conventional cell culture flatware or bioreactor culture [40]. Large and small EVs from either 20,000× *g* or 110,000× *g* ultracentrifugation steps were collected from both the VCaP and PC-3 lines cultured in either condition. EV samples were disassembled in acetonitrile and subjected to LC-ESI-MS with separation with either reversed-phase or hydrophilic columns. EVs were found to possess broadly similar physical characteristics regardless of the culture method, but significantly different levels of 459 metabolites were seen across both cell lines. Indeed, some molecules were unique to bioreactor or flatware culture, indicating different pathways of metabolic activity in the producing cells altering the identity of the resulting EVs. Whilst illustrative of the issues in EV sample generation, the implications of this work go beyond the differential metabolite results. There is significant interest in translating EVs as therapeutics to the clinic which will necessitate production scale-up to bioreactor levels from the flatware of standard research laboratories. Functional metabolite cargoes have already been proven in the case of cancer nutrient supply [33] and depending on the treatment modality of the candidate therapy, their efficacy may be impacted upon changes in metabolite content. This study should serve as a warning to therapy developers to characterise their products at all levels during process development. Moreover, understanding how the production conditions influence the metabolite content of the EVs may highlight novel methods to modulate their innate content.

## 4. Enrichment of Metabolites in EVs vs. EV Source

Metabolite enrichment in exosomes can sometimes facilitate the detection of certain low-abundance disease markers which are below the detection limit using the sample matrix alone. However, careful quantification of metabolite concentrations in both exosomes and matrices is required to validate enrichment. Failure to actively prove enrichment can have varied consequences. In the best-case scenario, it may be that a raw matrix shows biomarkers at sufficient concentrations to enable diagnosis, forgoing the need for advanced EV-targeted methods. However, it is more likely that a putative EV biomarker is not significantly enriched when compared to the biofluid, invalidating EV-associated biomarker application. More concerning outcomes are also possible. For the EV field, this is best exemplified by the contamination of EV preparations by exogenous nucleic acids from foetal bovine serum (FBS) media additives in a number of high-profile studies [56,57]. Prior to these cases, it was assumed that the ultracentrifugation-mediated EV-depletion of FBS was sufficient to remove excipient contaminants. Now, the findings of many publications have been called into question—a warning to all not to take EV sample preparation pitfalls lightly. Similarly, the recent identification of “exomeres” as distinct, functional nanoparticulate entities that can copurify with EV preparations may also present issues in future [58,59].

Therefore, it is important to always analyse the EV source matrix alongside the processed EV sample (e.g., the cell culture media direct from the bottle and without exposure to cells). For ultracentrifugation-based EV studies, retention and analysis of the supernatant can also suffice to determine any carryover from the cell culture media or biofluids, depending on the project. Alternatively, commercial isolation kits typically function by precipitation methods, and the work and supernatants from these should also be tested. A practical example comes from a comparative study on uEVs and platelet-derived EVs (pEVs) [45]. Therein, the metabolite content of uEVs purified by ultracentrifugation was measured by UHPLC-MS and compared to that of the originating urine filtrate, alongside pEVs compared with the parental platelets. In both systems, there was a high degree of metabolite overlap between the EVs and the source material, but certain classes of metabolites were enriched in the EV samples, suggesting an active recruitment of these molecules into the EVs. The largest changes in uEVs were observed for spermidine, ornithine and nicotinamide adenine dinucleotide (NAD), each enriched >600-fold compared to urine. Nucleotide and amino acid pathway metabolites were also significantly enriched. On the other hand, pEVs contained 11 unique molecules compared to the source platelets which only had one, but the enrichments were less dramatic for common metabolites. For example, a 250-fold upregulation of adenosine was the largest of these, followed by carnitine and various carnitine derivatives with 10- to 50-fold enrichments. Finally, inter-sample comparison of uEVs showed larger differences between the prostate cancer patients and the healthy controls than in the comparison between urine samples. Further comparison of the metabolite profiles from uEVs against pEVs revealed some common metabolites but also a broad range of unique molecules, highlighting the point that specific biofluids will be better starting points for specific diseases. Together, these data support the isolation of EVs as carriers of metabolites to identify otherwise missable biomarkers.

## 5. Metabolomics of Non-EV Samples

It is interesting to note how the metabolomics toolkit can be applied to questions of EV biology without direct EV analysis. This approach is well exemplified by complementary studies proving EVs as metabolically active entities by assaying the blood metabolome after incubation with EVs from rat primary hepatocyte culture [34,60]. Therein, small volumes of rat serum were incubated for 1 h with the EVs collected from 110,000× *g* ultracentrifugation and the metabolites then extracted through methanol–chloroform fractionation for UHPLC-MS analysis. In both studies, blood metabolome shifts were observed after incubation with EVs, specifically molecules of the arginine biosynthesis pathway, demonstrating that EVs are capable of actively modulating metabolites with their enzymatic content. This provides a novel concept of EVs as metabolic machines. These findings were further developed for arginase-positive EVs in an assay of pulmonary endothelial induction [34], based on the action of arginine as a nitric oxide precursor. The EVs inhibited the relaxation of isolated pulmonary arteries, providing direct proof that EVs can effect physiological changes through metabolome alteration. In related stories of active enzyme content, the presence of functional asparaginase, α2-6-sialyltransferase 1 (ST6Gal-I) and extracellular nicotinamide phosphoribosyltransferase (eNAMPT) have all been demonstrated in EVs [59,61,62]. eNAMPT is of particular note for its role in nicotinamide adenine dinucleotide metabolism, heavily associated with mechanisms of aging. Given the rapid rate of publication for these recent discoveries, it is exceedingly likely that metabolomics will reveal more such metabolically active EVs in future [63].

## 6. Conclusions and Future Directions

EV metabolomics is a nascent field but one with great potential, as evidenced by the interesting and varied findings presented in this review. The issues of EV sample preparation will always be inherent but are not insurmountable, as long as methods are clearly reported alongside sufficient controls and a suite of vesicle characterisation tests. That said, understanding in the field is continually evolving, as exemplified by the issues of contaminating nucleic acids from FBS media [57] or the disputed use of previously accepted EV markers [64]. To better facilitate inter-study comparison now and in posterity, full reporting is essential. It is worth consulting the MISEV and EV-TRACK (Transparent Reporting and Centralizing Knowledge in Extracellular Vesicle Research) guidelines in this regard [21,65]. These initiatives detail the minimal reporting requirements that should be included in a reproducible, quality study. However, there are no specific instructions for metabolomics reporting as yet, and these also need to be established or perhaps adopted from previous metabolomics standardisation initiatives [66]. EVpedia has allowances for metabolomics datasets [22], and we encourage researchers to fully utilise this resource and upload any findings to facilitate future bioinformatics analyses.

There is a great interest in using EVs for diagnostic purposes. However, the validation of biomarkers of any sort is a lengthy pursuit with many regulatory hurdles where failure is possible [67]. In these early days of EV metabolomics, it is worth pre-empting these challenges as much as possible through careful methodological planning. In this regard, it may be necessary to further standardise working practices. For example, the amount of EVs used for metabolomics varies across studies. One such work gives a vesicle count of 10^10^ particles for their experiments [45], and a similar number should perhaps be adopted by the field to ensure consistency between datasets. Moreover, the vast majority of studies presented here have used ultracentrifugation and the supremacy of this method as the “gold standard” has been challenged in recent years. With the ever-increasing amount of published methods, it is unlikely that a single best method will emerge and instead the choice of isolation method will be determined by the end application. Toward this end, we recommend that clinically relevant methods be adopted as soon as possible for biomarker studies [37,53]. Finally, the practical challenge of detecting biomarker shifts in a given patient is also worth considering. These may be too subtle to detect without previous patient sample data, necessitating a program of routine EV metabolite analysis that is unfeasible in practice. Future efforts should be made to establish a baseline EV metabolome for healthy humans from which deviations can be inferred.

EV metabolomics is useful for understanding certain EV functions, but questions remain regarding fundamental EV biology. For example, the investigation of EV subpopulation metabolomes has not been described, but may prove valuable to this area of high interest. This is a challenging task to undertake due the already low yield of total EV samples, particularly with the highly selective purification methods of marker-targeted affinity isolation or ultracentrifugation in combination with further chromatography steps [21]. Nonetheless, inevitable improvements in the sensitivity of metabolomics, such as nanoflow liquid chromatography-nanoelectrospray ionization (nLC-nESI) [68], along with more refined purification techniques, will hopefully enable these questions to be answered.

Despite the encouraging results collected herein, the full power of EV metabolomics is yet to be realized. We have used examples from biological pathologies, but the metabolome changes from physical stimuli can also be detected—as in the case of primates exposed to damaging radiation [46], which could potentially prove useful in monitoring exposure levels of radiotherapy technicians. Moreover, the metabolomics of pathogen EVs may further the understanding of infection from bacterial species [47]. Elsewise, the studies with patient-derived EVs presented here have focused on cancer, but metabolite biomarkers have already been suggested in neurodegeneration and even psychiatric disorders [69]. Such pathologies present different challenges in identifying valid patient–control cohorts but there is no reason that EV-targeted metabolomics may not prove useful in enriching the scanty biomarkers in these contexts. We hope that having read this review, metabolomics and EV researchers are encouraged to seek EV metabolite-related projects and contribute materially to this promising field.

## Figures and Tables

**Table 1:** Primary studies comprising metabolite analysis of EV samples.

Research Description	Sample Type, Source	EV Isolation Method	Metabolomics Workflow	Significant Metabolites
EVs secreted by cancerous cells contain a suite of metabolites that can be received and metabolised by cancer cells [33]	CD63+ EVs from cancer-associated fibroblasts	Total exosome isolation reagent	Methanol–chloroform fractionation of EVs followed by GC-MS or UHPLC	Pyruvate, citrate, glutamine, arginine, palmate, stearate
Plasma EVs from adenocarcinoma patients and PANC 1 cell culture shown to possess broad metabolomes [43]	CD63+/CD9+/TSG101+ EVs from human plasma EVs from PANC 1 cell culture	100,000× *g* ultracentrifugation	Methanol–chloroform fractionation of EVs followed by UHPLC-ESI-MS	Amino acids, substituted sugars
Metabolomics analysis of urinary EVs revealed potential prostate cancer biomarkers [32]	EVs from urine of either benign prostatic hyperplasia or prostate cancer patients	100,000× *g* ultracentrifugation	Methanol–chloroform fractionation of EVs followed by UHPLC-MS	Dehydroepiandrosterone sulphates, other androsterone sulphate isomers
Identified vesicular hexanal as a candidate chemoattractant for *Anopheles gambiae* vectors of malaria [44]	EVs from patient-derived red blood cell culture	110,000× *g* ultracentrifugation	GC-MS with headspace solid phase microextraction of EV samples	Hexanal, pentane2,2,4-trimethyl-pentane, 1,2,3-propanetriol diacetate
Comparative metabolomics of EVs from cells cultured with either conventional flatware or bioreactors revealed significant differences [40]	Large and small CD91+/CD9+/TSG101+ EVs from PC-3 and VCaP cell culture	20,000× *g* and 110,000× *g* ultracentrifugation	Acetonitrile dissolution of EVs followed by UHPLC/Q-TOF-MS with separation by either reverse phase or hydrophilic interaction	Amino acids, phosphatidylcholines, phosphatidylethanolamines, sphingomyelins
Metabolomics of urinary and platelet-derived EVs show enrichment of specific molecules compared to sample matrices [45]	CD9+/CD63+/TSG101+ EVs from urine of prostate cancer patients and platelet-derived EVs from matched serum samples	100,000× *g* ultracentrifugation for urinary EVs (uEVs) and 110,000× *g* for platelet-derived EVs	Acetonitrile dissolution of EVs followed by UHPLC-MS-MS	Spermidine, ornithine, carnitine derivatives, nicotinamide adenine dinucleotide, amino acids
Enrichment of certain metabolite classes detected in EVs after irradiation of rhesus macaques [46]	Plasma-derived CD63+ EVs from rhesus macaques	120,000× *g* ultracentrifugation	Acetonitrile dissolution of EVs followed by UHPLC/Q-TOF-MS	Carnitines, sphingomyelins, amino acids, 5-methycytosine, nonic acids
Outer membrane vesicles from toxigenic and nontoxigenic *Bacteroides fragilis* spp. exhibit different metabolomes [47]	Outer membrane vesicles from *Bacteroides fragilis* spp. culture	100,000× *g* ultracentrifugation	Cold methanol extraction of EV metabolites followed by UHPLC-MS or GC-MS	Creatinine, creatine, glycerate-2P, fumarate, malate, amino acids
Large EVs from atherosclerotic plaques present taurine enrichment [48]	EVs from human carotid atherosclerotic plaques	20,500× *g* ultracentrifugation	Proton nuclear magnetic resonance spectroscopy	Taurine, lactate, glycerophosphocholine
Change in serum EV metabolome after chemotherapy [49]	Serum exosomes from pancreatic cancer patients before and after chemotherapy	100,000× *g* ultracentrifugation	50% methanol and freeze–thaw cycle for extraction followed by LC-Q-TOF-MS	Alanyl-histidine, 6-dimethylaminopurine, leucyl-proline, and methionine sulfoxide
EVs secreted by mesenchymal stem cells contain a suite of metabolites that can be received and metabolised by other cells [50]	EVs derived from mesenchymal stem cell culture	Ultrafiltration followed by 110,000× *g* ultracentrifugation	Methanol extraction followed by CE-UV and HPLC-MS/MS	Diacylglycerols, sphingomyelins, lactate, glutamate
